# A hybrid-qudit representation of digital RGB images

**DOI:** 10.1038/s41598-023-39906-9

**Published:** 2023-08-22

**Authors:** Sreetama Das, Filippo Caruso

**Affiliations:** 1 Department of Physics and Astronomy, University of Florence, Via Sansone 1, Sesto Fiorentino, 50019 Italy; 2 European Laboratory for Non-Linear Spectroscopy (LENS), University of Florence, Via Nello Carrara 1, Sesto Fiorentino, 50019 Italy; 3 QSTAR and CNR-INO, Largo Enrico Fermi 2, 50125 Firenze, Italy

**Keywords:** Quantum information, Theoretical physics

## Abstract

Quantum image processing is an emerging topic in the field of quantum information and technology. In this paper, we propose a new quantum image representation of RGB images with deterministic image retrieval, which is an improvement over all the similar existing representations in terms of using minimum resource. We use two entangled quantum registers constituting of total 7 qutrits to encode the color channels and their intensities. Additionally, we generalize the existing encoding methods by using both qubits and qutrits to encode the pixel positions of a rectangular image. This hybrid-qudit approach aligns well with the current progress of NISQ devices in incorporating higher dimensional quantum systems than qubits. We then describe the image encoding method using higher-order qubit-qutrit gates, and demonstrate the decomposition of these gates in terms of simpler elementary gates. We use the Google Cirq’s quantum simulator to verify the image preparation in both the ideal noise-free scenario and in presence of realistic noise modelling. We show that the complexity of the image encoding process is linear in the number of pixels. Lastly, we discuss the image compression and some basic RGB image processing protocols using our representation.

## Introduction

State-of-the-art quantum computation, in both theoretical and experimental grounds, largely uses two-dimensional quantum systems known as Qubits. The same applies for the present-day noisy intermediate-scale quantum (NISQ) computing processors available for simulating real quantum circuits. Intrinsically, all the quantum systems designed as qubits have more than two energy levels, though the higher levels are maintained as non-functional. It is in principle possible to exploit these levels for quantum computation. Recently there has been a surge of interest to develop theoretical ideas and experimental tools to realize quantum computation using $d>2$ dimensional quantum systems, called Qudits^1–18^. This is usually referred to as ‘multi-valued quantum computation’. It is straightforward to observe that using qudits increases the storage capacity and reduces the complexity of a quantum circuit, improving the overall performance of a quantum information protocol. Experimental platforms, e.g. photonic systems^12,13^, trapped ions^4^, continuous spin systems^3,11^, nuclear magnetic resonance^9,10^, molecular magnets^2^ and transmon qudits^19^ has been used to test qudit-based computation. The present literature on qudits has shed light on to the construction of elementary gates for quantum circuits where all the qudits have same dimension^1,7,20–24^. However, quantum circuit composed of qudits of different dimensions, often termed as ‘hybrid-qudit system’, remain relatively unexplored^25–27^.

Quantum computation has found its application in a plethora of technological fields, quantum image processing being one of them. It is a rapidly developing topic with potential applications in space science, medicine, automobile engineering etc. Compared to classical bits, one needs logarithmically less number of qubits to store the same amount of classical information. Additionally, quantum properties like superposition and entanglement leads to exponential quantum speed-up of the image processing algorithms^28,29^. The first step of an image processing algorithm is to encode the classical image as a quantum state. A number of different forms of quantum encoding methods have been proposed^29–35^. They differ in their applicability for binary, greyscale or color images, the amount of resources consumed, and the efficiency of the image retrieval process. For binary and greyscale images, Flexible representation of quantum images (FRQI)^30^ and Novel-enhanced quantum representation of images (NEQR)^32^ are the most commonly used encoding methods in the current literature. Compared to FRQI, NEQR employs higher number of qubits to encode the greyscale pixel values and has a more accurate image retrieval process, which we will discuss in the upcoming sections. For color images, specifically those having an RGB color channel format, several quantum representations inspired from FRQI and NEQR have been proposed. To mention some of them, ref.^31^ extends the FRQI method to encode the color channel intensities using three qubits, though it suffers the same drawback as FRQI for image retrieval. On the other hand, the NEQR-based color image encoding methods^33,35^ use 24 qubits to encode the colors, and have accurate image retrieval. In ref.^34^, the authors show that the number of necessary qubits can be reduced to 10 for encoding the colors, while retaining the accurate image retrieval.

In this work, we aim to reduce the number of quantum units required to encode an RGB image, while adhering to the accurate image retrieval process. As it has been shown in^36^, following the same approach as NEQR, and replacing the qubits with qutrits, only 6 qutrits are required to encode the greyscale values varying from 0 to 256. This means that the encoding of RGB color channel intensities should take 16 qutrits, which is still pretty large. As we propose here, it is possible to encode an RGB image using only 7 qutrits. Using higher than three dimensional qudits can logarithmically reduce the required resources. Interestingly, in ref.^37^, it is argued that systems with three basis states indeed construct the most cost-effective circuit compared to $n\ne 3$ dimensional systems^36^. In the IBMQ platform, one can excite the third energy level of a superconducting (SC) qubit and convert it to a qutrit. The Google Cirq quantum computing platform^38^ provides quantum simulation using qudits and corresponding gates. Rigetti’s quantum computation supports the activation, gate implementation and measurement on a real qutrit^39^. All the above imply that the qutrit-based quantum computing is imminent, and these motivate us to use qutrits in quantum representation of images.

Our encoding scheme also explores the possibility of using both qubits and qutrits to encode the pixel position and color information, thus giving rise to a hybrid qubit-qutrit circuit. We call this a Hybrid Qudit Quantum Representation (HQDQR) of RGB images. The elementary gates of a hybrid-qudit system has been discussed before^25,26^. In this manuscript, we show the decomposition of higher-order hybrid qudit gates in terms of elementary single-qudit and hybrid two-qudit gates. This in turn shows that, the number of elementary gates needed for our proposed image encoding is much less compared to the other existing RGB image representations. Thus, HQDQR uses minimum number of quantum units as well as minimum number of gates to encode an RGB image. The complexity of the image encoding process is linear in the number of pixels.

The paper is organized as follows. In Section “Methods” we revisit some elementary gates of qutrit and hybrid qubit-qutrit quantum circuit, and show their decomposition in terms of elementary single-qudit and two-qudit gates. In Section “Model” we briefly discuss the existing RGB image representations. Following that we introduce our hybrid qudit representation, calculate the complexity of the image encoding process and discuss the image compression. Additionally, we present some basic RGB image operations using our image representation. In Section “Experiment”, we present the results of testing the image encoding and retrieval in the Cirq quantum simulator. Finally, we conclude in section “Discussion”.

## Methods

In this section, we present preliminary concept of quantum gates used in a quantum circuit constituted of qudits, which will be useful in order to describe our image representation. The gates used in a quantum circuit apply an unitary transformation on the input state. In case of qubits, any unitary transformation can be asymptotically achieved by repeatedly applying a set of single and two-qubit elementary gates, allowing a certain amount of error. These elementary gates are called *universal gates* for qubits. The idea of universality can be extended to qudits. In fact a number of works have proposed the universal gates in a qudit circuit^1,7,20,22^. However, the mathematical idea of universality may not always be suitable to apply for physically realizing a complex quantum circuit. In practice, the gates used to achieve a unitary operation on any number of qudits should be easy to realize experimentally and should have hight fidelity, so that it can be implemented efficiently in a real quantum hardware. There has been a number of experimental proposals to realize basic qudit gates in laboratory.

The above works assume that the all the qudits in a circuit has the same dimension *d*. In principal, it is possible to build a circuit with different dimensional qudits. A few works discuss the basic gates in a hybrid qudit circuit, and quantum computation using such systems^25,26^.

### Quantum computation in qutrit systems

The Hilbert space of a three-level quantum system or Qutrit is spanned by the orthogonal basis vectors $\{|0\rangle , |1\rangle , |2\rangle \}$. The elementary and universal qutrit gates, and the possibility of their physical realization, have been investigated^1,20,22,40–43^.

### Single qutrit gates

#### Ternary bit-flip gates:

In analogy to the qubit bit-flip or *X* gate, the ternary *X* gate flips the basis states of a qutrit.

There are six ternary *X* gates as listed below^36,40^,

1 $$ \begin{gathered}   \sigma _{{ + 0}}^{x}  = \left( {\begin{array}{*{20}c}    1 & {\quad 0} & {\quad 0}  \\    0 & {\quad 1} & {\quad 0}  \\    0 & {\quad 0} & {\quad 1}  \\   \end{array} } \right),\sigma _{{ + 1}}^{x}  = \left( {\begin{array}{*{20}c}    0 & {\quad 0} & {\quad 1}  \\    1 & {\quad 0} & {\quad 0}  \\    0 & {\quad 1} & {\quad 0}  \\   \end{array} } \right), \hfill \\   \sigma _{{ + 2}}^{x}  = \left( {\begin{array}{*{20}c}    0 & {\quad 1} & {\quad 0}  \\    0 & {\quad 0} & {\quad 1}  \\    1 & {\quad 0} & {\quad 0}  \\   \end{array} } \right),\sigma _{{01}}^{x}  = \left( {\begin{array}{*{20}c}    0 & {\quad 1} & {\quad 0}  \\    1 & {\quad 0} & {\quad 0}  \\    0 & {\quad 0} & {\quad 1}  \\   \end{array} } \right), \hfill \\   \sigma _{{12}}^{x}  = \left( {\begin{array}{*{20}c}    1 & {\quad 0} & {\quad 0}  \\    0 & {\quad 0} & {\quad 1}  \\    0 & {\quad 1} & {\quad 0}  \\   \end{array} } \right),\sigma _{{02}}^{x}  = \left( {\begin{array}{*{20}c}    0 & {\quad 0} & {\quad 1}  \\    0 & {\quad 1} & {\quad 0}  \\    1 & {\quad 0} & {\quad 0}  \\   \end{array} } \right) \hfill \\  \end{gathered}  $$

**Figure 1:**
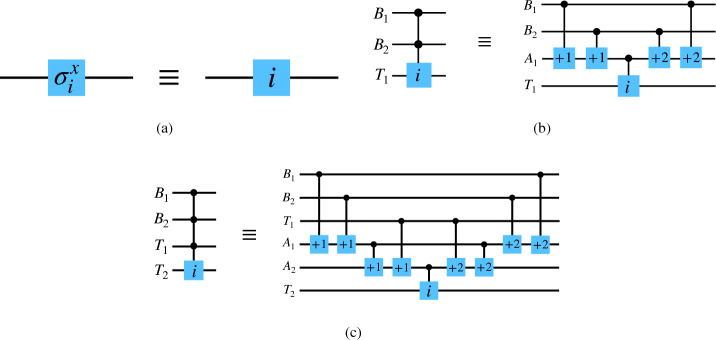
(**a**) The notation we use for qutrit bit-flip gates in our circuit diagrams, where $i \in \{+0, +1, +2, 01, 12, 02\}$. (**b**) Decomposition of a three-qudit hybrid Toffoli gate. (Left) A hybrid Toffoli gate where the control is on two qubits $\{B_{1}, B_{2}\}$ and the target is a qutrit $T_{1}$. (Right) The decomposition of the gate using an auxiliary qutrit $A_{1}$ and hybrid controlled *X* gates. (**c**) Decomposition of a four-qudit hybrid Toffoli gate. (Left) A hybrid Toffoli gate where the control is on $\{B_{1}, B_{2}\}$ and $T_{1}$, and the target qutrit is $T_{2}$. (Right) The decomposition of the gate using two auxiliary qutrits $\{A_{1}, A_{2}\}$ and hybrid controlled *X* gates.

The first one is identity operator which does not change anything. The operators $\{\sigma ^{x}_{+1}, \sigma ^{x}_{+2}\}$ transform the basis $|x\rangle$ by $|x\rangle \rightarrow |(x+1)\mod 3\rangle$ and $|x\rangle \rightarrow |(x+2)\mod 3\rangle$ respectively, and work on all the basis states simultaneously. Lastly, $\{\sigma ^{x}_{01}, \sigma ^{x}_{12}, \sigma ^{x}_{02}\}$ swaps the two basis states $\{|i\rangle ,|j\rangle \}$ in the subscript of $\sigma ^{x}_{ij}$, while leaving the third basis state unchanged. Following earlier works, we will use a simple notation for the above gates in the circuit diagrams which is shown in Fig. 1a.

#### Ternary Hadamard gate:

The Hadamard gate $H_{2}$ in a two-dimensional Hilbert space ${\mathscr {H}}_{2}$ is the quantum Fourier transform of the computational basis to the eigenbasis of Pauli matrix $\sigma ^{x}$. In an analogous way, the ternary Hadamard gate $H_{3}$ in the Hilbert space ${\mathscr {H}}_{3}$ can be defined as the following,

2 $$\begin{aligned} H_{3} = \frac{1}{\sqrt{3}}\begin{pmatrix} 1 &{} \quad 1 &{} \quad 1\\ 1 &{} \quad e^{i\frac{2 \pi }{3}} &{} \quad e^{-i\frac{2 \pi }{3}}\\ 1 &{} \quad e^{-i\frac{2 \pi }{3}} &{} \quad e^{i\frac{2 \pi }{3}} \end{pmatrix}. \end{aligned}$$

An experimental realization of this gate has been possible^41^ using superconducting qutrits.

### Two-qutrit gates

#### *Ternary controlledXgates:*

A binary controlled *X* gate is a two-qubit gate that performs bit-flip operation *X* on the target qubit, only if the control qubit is in state $\vert 1\rangle$. The above is the standard notion, although it can be configured so that the target qubit is flipped only when the control qubit is in state $|0\rangle$. In a qutrit, there are 18 different generalized controlled *X* operations (three possible control states and six target flips for each of them), each of them being a $9\times 9$ unitary matrix. As an example, if we want to flip the target qutrit state from $|0\rangle$ to $|1\rangle$ when the control qutrit state is $|2\rangle$, the corresponding unitary will be,

3 $$ \begin{gathered}   U = (|0\rangle \langle 0| + |1\rangle \langle 1|) \otimes {\mathbb{I}}_{3}  + |2\rangle \langle 2| \otimes \sigma _{{01}}^{x}  \hfill \\   \quad = \left( {\begin{array}{*{20}c}    1 & {\quad 0} & {\quad 0} & {\quad 0} & {\quad 0} & {\quad 0} & {\quad 0} & {\quad 0} & {\quad 0}  \\    0 & {\quad 1} & {\quad 0} & {\quad 0} & {\quad 0} & {\quad 0} & {\quad 0} & {\quad 0} & {\quad 0}  \\    0 & {\quad 0} & {\quad 1} & {\quad 0} & {\quad 0} & {\quad 0} & {\quad 0} & {\quad 0} & {\quad 0}  \\    0 & {\quad 0} & {\quad 0} & {\quad 1} & {\quad 0} & {\quad 0} & {\quad 0} & {\quad 0} & {\quad 0}  \\    0 & {\quad 0} & {\quad 0} & {\quad 0} & {\quad 1} & {\quad 0} & {\quad 0} & {\quad 0} & {\quad 0}  \\    0 & {\quad 0} & {\quad 0} & {\quad 0} & {\quad 0} & {\quad 1} & {\quad 0} & {\quad 0} & {\quad 0}  \\    0 & {\quad 0} & {\quad 0} & {\quad 0} & {\quad 0} & {\quad 0} & {\quad 0} & {\quad 1} & {\quad 0}  \\    0 & {\quad 0} & {\quad 0} & {\quad 0} & {\quad 0} & {\quad 0} & {\quad 1} & {\quad 0} & {\quad 0}  \\    0 & {\quad 0} & {\quad 0} & {\quad 0} & {\quad 0} & {\quad 0} & {\quad 0} & {\quad 0} & {\quad 1}  \\   \end{array} } \right), \hfill \\  \end{gathered}  $$

where $\mathbb {I}_{d}$ is a *d*-dimensional identity operator. The rest 17 unitaries can be constructed in a similar way.

### Quantum computation in hybrid qubit-qutrit systems

In a hybrid quantum system constituted of both qubits and qutrits, the single qudit gates remain unchanged. However, there can exist two or multi-qudit gates which act on a composite system of qubits and qutrits.

#### Two-qudit hybrid gates

#### *Hybrid controlledXgate:*

For this class of gates, the control can be a qubit and the target can be a qutrit, or the vice versa. Suppose, if the control qubit is in state $|1\rangle$, $\sigma ^{x}_{12}$ is applied on the target qutrit. The corresponding unitary is,

4 $$ \begin{gathered}   U = |0\rangle \langle 0| \otimes {\mathbb{I}}_{3}  + |1\rangle \langle 1| \otimes \sigma _{{12}}^{x}  \hfill \\   \quad  = \left( {\begin{array}{*{20}c}    1 & {\quad 0} & {\quad 0} & {\quad 0} & {\quad 0} & {\quad 0}  \\    0 & {\quad 1} & {\quad 0} & {\quad 0} & {\quad 0} & {\quad 0}  \\    0 & {\quad 0} & {\quad 1} & {\quad 0} & {\quad 0} & {\quad 0}  \\    0 & {\quad 0} & {\quad 0} & {\quad 1} & {\quad 0} & {\quad 0}  \\    0 & {\quad 0} & {\quad 0} & {\quad 0} & {\quad 0} & {\quad 1}  \\    0 & {\quad 0} & {\quad 0} & {\quad 0} & {\quad 1} & {\quad 0}  \\   \end{array} } \right) \hfill \\  \end{gathered}  $$

On the other hand, if the control is on the qutrit and the target is a qubit, and the qubit state flips when the control qutrit is in state $|2\rangle$, then the unitary can be expressed as,

5 $$ \begin{gathered}   U = (|0\rangle \langle 0| + |1\rangle \langle 1|) \otimes {\mathbb{I}}_{2}  + |2\rangle \langle 2| \otimes \sigma ^{x}  \hfill \\   \quad  = \left( {\begin{array}{*{20}c}    1 & {\quad 0} & {\quad 0} & {\quad 0} & {\quad 0} & {\quad 0}  \\    0 & {\quad 1} & {\quad 0} & {\quad 0} & {\quad 0} & {\quad 0}  \\    0 & {\quad 0} & {\quad 1} & {\quad 0} & {\quad 0} & {\quad 0}  \\    0 & {\quad 0} & {\quad 0} & {\quad 1} & {\quad 0} & {\quad 0}  \\    0 & {\quad 0} & {\quad 0} & {\quad 0} & {\quad 0} & {\quad 1}  \\    0 & {\quad 0} & {\quad 0} & {\quad 0} & {\quad 1} & {\quad 0}  \\   \end{array} } \right), \hfill \\  \end{gathered}  $$

where $\sigma ^{x}$ is the qubit *X* gate. This unitary is same as that in Eq. (4), but as we will see in the upcoming subsections, this may not always be the case.

#### Multi-qudit hybrid gates

##### *Hybrid Toffoli gates:*

Now suppose that there are one control qubit and one control qutrit, while the target is a qutrit. If the control qudits are respectively in state $|1\rangle$ and $|2\rangle$, the target qutrit undergoes the bit-flip operation $\sigma ^{x}_{12}$. This is analogous to the Toffoli gate or controlled controlled X gate for qubits, and we will call it *hybrid Toffoli gate* in this paper. It can be decomposed using simpler single-qudit and hybrid two-qudit gates. We show such a decomposition in Fig. 1b, where there are two control qubits and a target qutrit. The decomposition uses one auxiliary qutrit. If there are *n* control qudits, we will need $(n-1)$ auxiliary qutrits. The last two hybrid gates are applied on the auxiliary qutrit to bring it back in the initial state $|0\rangle$, so that it can be used for the decomposition of another gate. In Fig. 1c, we show the decomposition of a higher order hybrid Toffoli gate in which the control is on two qubits and a qutrit, and the target is a qutrit.

###### Theorem 1

The complexity of a hybrid Toffoli gate with *n* number of control qubits and qutrits is $4n-3$.

###### Proof

There are *n* controlled *X* gates from *n* control qudits to the auxiliary qutrits, and total $n-2$ controlled *X* gates among all the adjacent pairs of auxiliary qutrits. The same number of gates are applied to bring back the auxiliary qutrits in state $|0\rangle$. There is one controlled *X* gate between the last auxiliary qutrit and the target qudit. So, the total number of gates used is $2(n+(n-2))+1=4n-3$. $\square$

##### *Generalized hybrid Toffoli gates:*

**Figure 2:**
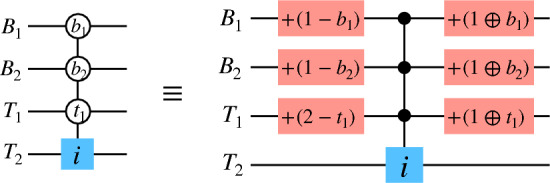
Decomposition of a higher order generalized Toffoli gate. (Left) The control qudits are two qubits $\{B_{1}, B_{2}\}$ and a qutrit $T_{1}$, and the target is a qutrit $T_{2}$. The target qutrit undergoes $\sigma _{i}^{x}$ when the control qudits are in states $|b_{1}\rangle$, $|b_{2}\rangle$ and $|t_{1}\rangle$ respectively. (Right) The decomposition of this gate in terms of $\sigma ^{x}_{i}$s and four-qudit hybrid Toffoli gate.

For the case discussed above, we assumed that for a control qubit and a control qutrit, the Toffoli gate is activated when the control states are respectively $|1\rangle$ and $|2\rangle$, i.e. when the control qudits are in their highest state. In principal, the gates can be designed to be activated for any of the control states $\{|0\rangle , |1\rangle ,..., |d-1\rangle \}$ of a qudit. We call such gates *generalized hybrid Toffoli gates*. The decomposition of a generalized hybrid Toffoli gate is shown in Fig. 2, where 2*k* single qudit gates, *k* being the number of controls with generalized bit-values, and a higher order hybrid Toffoli gate have been used. The later can be further decomposed in terms of the hybrid two-qudit gates using auxiliary qutrits as shown in Fig. 1c.

###### Lemma 1

The complexity of a generalized higher order hybrid Toffoli gate with *n* control qubits and qutrits is $4n-3+2k$. The maximum value is $6n-3$, reached when $k=n$.

**Figure 3:**
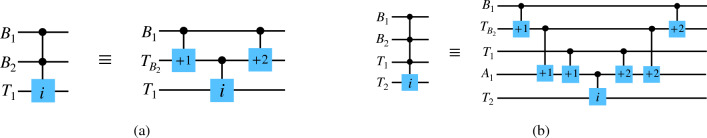
The improved gate decomposition using effective qutrits. (**a**) The decomposition of the circuit in Fig. 1b where the qubit $B_{2}$ becomes effective qutrit $T_{B_{2}}$. (**b**) The decomposition of the circuit in Fig. 1c, where now one auxiliary qubit $A_{1}$ is required.

In^44^, the authors demonstrate that the higher order Toffoli gates for qubits can be synthesized without any auxiliary quantum system, if the third energy levels of these qubits are activated. This extra energy level is used only in the intermediate steps to store information, while the input and the output of the circuit remains in the same dimensional Hilbert space. The authors also show that such a circuit has depth $\log N$ for synthesizing a higher order Toffoli gate acting on *N* qubits. Clearly, the decomposition of a hybrid qubit-qutrit gate can facilitate from a lower circuit depth and less number of elementary gates by using effective qutrits in place of qubits. Such a decomposition is shown in Fig. 3.

###### Theorem 2

The complexity of a hybrid Toffoli gate with $n_{1}$ control qubits ( or, $n_1 -1$ effective qutrits ) and $n_{2}$ control qutrits is $2n+2n_{2}-1$, where $n=n_1 + n_2$.

###### Proof

As evident from Fig. 3b, the number of auxiliary qutrits needed in this case is $n_{2}$. There are $n_{1}-1$ controlled *X* gates between $n_{1}$ control qubits, and 2 controlled *X* gates for each of the $n_{2}$ qutrit-auxiliary qutrit pair. Thus, the total number of gates is $2(n_{1}-1+2n_{2})+1=2n+2n_{2}-1$. $\square$

The improvement in the number of gates by using effective qutrits is $4n-3-(2n+2n_{2}-1)=2n_{1}-2$.

###### Lemma 2

The complexity of a generalized higher order hybrid Toffoli gate with $n_{1}$ control qubits ($n_1 -1$ effective qutrits) and $n_2$ control qutrits is $2n+2n_{2}-2+2k$.

## Model

Equipped with the necessary tools to design hybrid qubit-qutrit circuit, in this section, we describe our proposed RGB image representation. Before that we briefly discuss some of the existing RGB image representations, to make a clear comparison of our representation with the existing approaches. We do not discuss here the angular representation like FRQI, and stick to the encoding methods corresponding to deterministic image retrieval which is relevant for our work.

### Multi-channel representation for images on quantum computers (MCQI)

In this representation^31^, a $2^{n}\times 2^{n}$ dimensional RGB image is encoded using $2n+3$ qubits. The first 3 qubits encode the intensities of the three channels $\{\textrm{R, G, B}\}$ and the rest 2*n* qubits encode the positions. The intensities of the three channels are encoded using the angles $\theta _{i}$ ($i=R,G,B$). The quantum state corresponding to the image is the following,

6 $$\begin{aligned} |I\rangle =&\frac{1}{2^{n}}\sum \limits _{Y=0}^{2^{n}-1}\sum \limits _{X=0}^{2^{n}-1} (\cos \theta ^{i}_{R}|000\rangle + \cos \theta ^{i}_{G}|001\rangle + \cos \theta ^{i}_{B}|010\rangle \nonumber \\&+ \sin \theta ^{i}_{R}|100\rangle + \cos \theta ^{i}_{G}|101\rangle + \cos \theta ^{i}_{B}|110\rangle \nonumber \\&+\cos 0|011\rangle + \sin 0|111\rangle )\times |YX\rangle , \end{aligned}$$

where $|X\rangle$ and $|Y\rangle$ are the $2^{n}$ basis states of an *n*-qubit system, and $\theta _{i}$ lies in the range $[0, \pi /2]$. The 3-qubit register has 8 basis states, from which six are used to encode the color information, and the coefficients corresponding to $|011\rangle$ and $|111\rangle$ are set as constants $\sin 0$ and $\cos 0$, so that they do not carry any information. To retrieve the images, one has to perform repeated measurements on all the three color qubits to probabilistically obtain the coefficients $\cos \theta _{i}$ and $\sin \theta _{i}$. Because of the probabilistic nature of the output, such an image retrieval is not accurate, and large number of measurements are required to reach a particular accuracy.

### Novel quantum representation of color digital images (NCQI)

This encoding method^33^ is inspired from NEQR, where the intensities of each channel is encoded using the basis vectors of 8 qubits. So, in total there are 24 qubits needed to encode the color of a pixel. For a $2^{n}\times 2^{n}$ dimensional image, the positions are encoded using 2*n* qubits. So, the total number of qubits in $2n + 24$. The quantum state corresponding to a $2\times 2$ image looks like the following,

7 $$\begin{aligned} |I\rangle =\frac{1}{2^{n}} \sum \limits _{Y=0}^{2^{n}-1}\sum \limits _{X=0}^{2^{n}-1}|R_{XY}\rangle |G_{XY}\rangle |B_{XY}\rangle |YX\rangle , \end{aligned}$$

where $|C_{XY}\rangle =|C^{q-1}_{XY}...C^{1}_{XY}C^{0}_{XY}\rangle$, $C=\{R, G, B\}$ and $q=8$. A projective measurement on the position and the color qubits can accurately retrieve the pixel positions and corresponding pixel colors and intensities. However, one should note that while using the real quantum processors for retrieving the images, one still has to perform a finite number of repeated measurements to obtain all the basis vectors corresponding to the intensities.

The time complexity of preparing quantum images using NCQI is quadratically less compared to that using MCQI. This advantage is analogous to the advantage of NEQR over FRQI. Also, NCQI allows for more complex color transformations, and solves the problem of probabilistic retrieval of pixel values. A limitation of both the above representations is that they consider only square images. To generalize this for rectangular images, an improved encoding method has been proposed^35^.

### Optimized quantum representation of color images

In this encoding method^34^, the color information is stored using two entangled quantum registers. The first register with two qubits encodes the channel information, while the second register with eight qubits encodes the intensity of that channel. The pixel positions are again encoded using 2*n* qubits for a $2^{n}\times 2^{n}$ classical image. The quantum image state is,

8 $$\begin{aligned}&|I\rangle = \frac{1}{2^{n}}\Big ( |R_{XY}\rangle |00\rangle +|G_{XY}\rangle |01\rangle + |B_{XY}\rangle |10\rangle \nonumber \\ {}&\qquad+ |S_{XY}\rangle |11\rangle \Big ) \otimes |YX\rangle , \end{aligned}$$

where $|C_{XY}\rangle$ $(C=R, G, B)$ is the same as defined for NCQI. This representation uses only 10 qubits to encode the colors, which is a significant improvement over NCQI.

**Figure 4:**
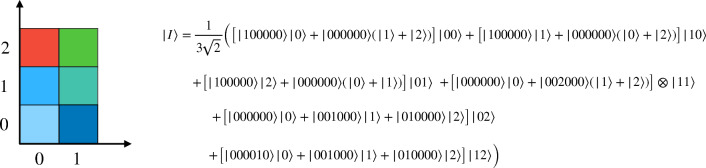
A $3\times 2$ dimensional RGB image and its corresponding quantum image state $|I\rangle$ represented using HQDQR.

**Figure 5:**
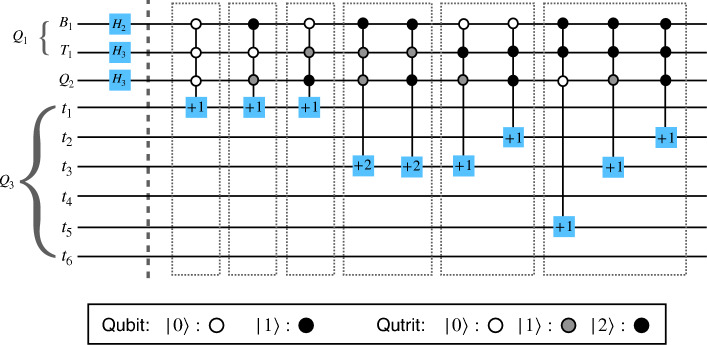
The circuit for encoding the quantum image state $\vert I\rangle$ in Fig. 4. The register $Q_{1}$ constituted of one qubit $B_{1}$ and one qutrit $T_{1}$ encodes the pixel positions. The qutrit $Q_{2}$ encodes color channels, and the register $Q_{3}$ with six qutrits $t_{i}$ ($i=0, 1,.., 5$) encodes the intensity of the color channels. The six blocks with thin outlines stand for encoding steps for six pixel positions.

### Hybrid-qudit representation of color images (HQDQR)

Recently there has been a proposal of a novel qutrit representation of greyscale images^36^, in which both the pixel positions and pixel values are encoded using qutrits. Compared to 8 qubits in NEQR, it needs at least 6 qutrits to encode the 256 shades of grey. Since $$3^6 > 256 $$, a number of the energy levels remain redundant, which can be used for error correction. In this work, we show that a total of 7 qutrits are required to encode the color information of an RGB image. The color channel $$\{\textrm{R,G,B}\}$$ can be encoded using the three levels of a qutrit. With this qutrit, a register of 6 qutrits is entangled, which encodes the information about the intensity of each color. Additionally, we consider rectangular images instead of square ones, for which the pixel positions can be encoded using quantum registers constituted of only qubits or qutrits, or both where it is appropriate.

First, we consider the most general case of an $M \times N$ dimensional classical image such that $M=3^{m}$ and $N=2^{n}$. The initial quantum state of all the three registers is,

9 $$\begin{aligned} |\Psi _{0}\rangle = \underbrace{|000000\rangle }_\text {6 qutrits} \otimes \underbrace{|0\rangle }_\text {qutrit} \otimes \underbrace{|000...0\rangle }_\text {m+n qudits} \end{aligned}$$

Now, Hadamard operator $H_{3}^{\otimes m} \otimes H_{2}^{\otimes n}$ is applied on the qudits of the last register to transform the state of this register to a fully superposed state.

10 $$\begin{aligned} |\Psi _{1}\rangle = \frac{1}{\sqrt{2^{n}3^{m}}}\sum \limits _{Y=0}^{2^{n}}\sum \limits _{X=0}^{3^{m}} |000000\rangle \otimes |0\rangle \otimes |YX\rangle . \end{aligned}$$

Now, ${\mathscr {H}}_{3}$ is applied on the second register to prepare the following state,

11 $$\begin{aligned} |\Psi _{2}\rangle = \frac{1}{\sqrt{2^{n}3^{m+1}}}\sum \limits _{Y=0}^{2^{n}}\sum \limits _{X=0}^{3^{m}}|000000\rangle \otimes (|0\rangle + |1\rangle + |2\rangle ) \otimes |YX\rangle . \end{aligned}$$

We assume that the states $|0\rangle$, $\vert 1\rangle$ and $\vert 2\rangle$ represent respectively the color channels R, G and B. In the next step, for each pixel position $|YX\rangle$ and for each color channel, a number of controlled *X* operations is used to flip the qutrits in the first register, where the control lies on the qudits in the first and second register. We can express this operation as,

12 $$\begin{aligned} |\Psi _{3}\rangle &= \frac{1}{\sqrt{2^{n}3^{m+1}}}\sum \limits _{Y=0}^{3^{m}}\sum \limits _{X=0}^{2^{n}} \Omega _{XY} \big ( |000000\rangle \otimes (|0\rangle + |1\rangle \nonumber \\ &\quad + |2\rangle )\otimes |YX\rangle \big ) \nonumber \\& =\frac{1}{\sqrt{2^{n}3^{m+1}}}\sum \limits _{Y=0}^{3^{m}}\sum \limits _{X=0}^{2^{n}} (|R_{XY}\rangle |0\rangle + |G_{XY}\rangle |1\rangle + |B_{XY}\rangle |2\rangle ) \nonumber \\ &\quad \otimes |YX\rangle , \end{aligned}$$

where $\Omega _{XY}=\bigotimes \limits _{i=0}^{5}\Omega _{XY}^{i,C}$, $|C_{XY}\rangle = |C^{5}_{XY}C^{4}_{XY}..C^{0}_{XY}\rangle$, $C=\{R, G, B\}$, and each $\Omega _{XY}^{i,C}$ is a higher order generalized hybrid Toffoli gate flipping the $i^{\textrm{th}}$ qubit of the color channel *C*, in position $|YX\rangle$. Equation (12) is our desired quantum representation. As an example, we consider a $3\times 2$ dimensional RGB image shown in Fig. 4 and its corresponding quantum image state $|I\rangle$. The different steps for encoding this image are shown in Fig. 5, where each block surrounded by thin rectangular border stands for one particular pixel position. Thus, by the full use of quantum superposition and entanglement, in place of 24 qubits needed to store the RGB color information, we need only 7 qutrits, when enough auxiliary qutrits are available. Note that, this encoding method works whenever $M \times N \le 2^{n}\times 3^{m}$, for which the extra basis states in Eq. (10) corresponds to extra pixels in the quantum image for which all three color channels have intensity 0.

### Special cases

Above we considered both qubits and qutrits to encode the pixel positions of a rectangular image. However, depending on the dimension of the image, it might be optimum to use only qubits or only qutrits to encode the positions, to minimize the redundancy in the number of energy levels. In this following subsection, we discuss the quantum representation for RGB images in such cases.

#### All-qubit third register

Suppose the dimension of an image is $M\times N$ such that $M=2^{m}$ and $N=2^{n}$. The quantum image state in this case becomes,

13 $$\begin{aligned}&|I\rangle = \frac{1}{\sqrt{2^{m+n}}}\sum \limits _{Y=0}^{2^{m}}\sum \limits _{X=0}^{2^{n}} (|R_{XY}\rangle |0\rangle + |G_{XY}\rangle |1\rangle \nonumber \\&\quad+ |B_{XY}\rangle |2\rangle ) \otimes |YX\rangle . \end{aligned}$$

**Figure 6:**
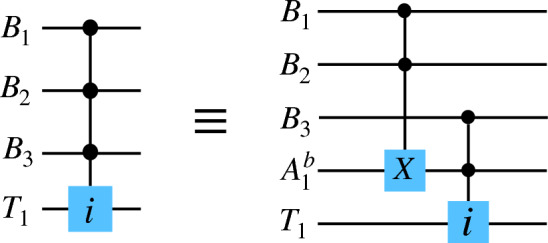
Decomposition of a higher order hybrid Toffoli gate where the control qudits $\{B_{1}, B_{2}, B_{3}\}$ are all qubits. The decomposition is shown in terms of auxiliary qubit $A^{b}_{1}$, Toffoli gate, and hybrid Toffoli gate.

The generalized higher order Toffoli gates now has only qubits as the control, and a qutrit as the target. It is possible to decompose such gates using auxiliary qutrits as shown in Fig. 1, and using effective qutrits as in Fig. 3. One can also use auxiliary qubits, the decomposition then can be obtained in terms of Toffoli gates and hybrid Toffoli gates, as shown in Fig. 6. Of course, one can use an extra auxiliary qubit to decompose the Toffoli gate in Fig. 6 into controlled-*X* gates. We choose to use Toffoli gate since it is already known that a higher order Toffoli gate with *p* ($p>2$) controls, can be decomposed into $4p-8$ Toffoli gates, when $p-2$ auxiliary qubits are present^45^.

#### All-qutrit third register

If the image dimension is such that $M=3^{m}$ and $N=3^{n}$, the quantum image state becomes,

14 $$\begin{aligned}&|I\rangle = \frac{1}{\sqrt{3^{m+n}}}\sum \limits _{Y=0}^{3^{m}}\sum \limits _{X=0}^{3^{n}} (|R_{XY}\rangle |0\rangle + |G_{XY}\rangle |1\rangle \nonumber \\& \quad+ |B_{XY}\rangle |2\rangle ) \otimes |YX\rangle . \end{aligned}$$

The generalized higher order Toffoli gates acts only on qutrits in this case, both as control and target. We do not show the decomposition of these gates, because it can be obtained in the same way as shown in Figs. 1 and 2.

**Table 1:** The number of elementary gates (one and two-qudit gates) used for different RGB image encoding methods

Representation	Elementary gates used
MCQI	$24\times 2^{4n}-9\times 2^{2n} + 2n + 2$
NCQI	$2n + 24\times 2^{2n}\times 48(n-1)$
OCQR	$2n+2+24\times 2^{2n} \times 48n$
HQDQR	$m+n+1+18(2^{n}\times 3^{m})(6(m+n)+3)$

For the first three rows the number of pixels in the image is $2^{2n}$, and for the last row the same is $2^{n} \times 3^{m}$.

### Complexity of the encoding method

We analyze the complexity of the quantum image encoding method in two steps.

1. There are $m+n+1$ single qudit gates applied in the first step to encode the pixel positions and color channels, the complexity of which is ${\mathscr {O}}(m+n+1)$.
2. There are $2^{n}\times 3^{m}$ pixels, each pixel having three channels. For each channel, one needs to flip maximum 6 qutrits to encode the intensity. So the maximum number of generalized higher order hybrid Toffoli gates required for each channel is 6. Now, a generalized higher order hybrid Toffoli gate with $m+n+1$ control qudits can be decomposed using maximum of $(6(m+n+1)-3)$ elementary gates. So the total complexity of this step is no more than $(18(2^{n}\times 3^{m})(6(m+n+1)-3)$ which is ${\mathscr {O}}(N)$, i.e. linear in the number of pixels.

So, the total complexity of the image encoding process is ${\mathscr {O}}(N)$. It is to be noted that, though the NCQI and OCQR encoding method discussed earlier has similar order of complexity, the number of elementary gates used for encoding is much less for HQDQR. A comparison between different RGB image representations and the number of elementary gates used has been presented in Table 1. From the table, it is easy to see that, the total number of pixels $2^{2n}$ or $2^{n}\times 3^{m}$ remains common for all complexity values, but the multiplication factor is smallest for HQDQR.

Let us compare the depth of the HQDQR encoding circuit with the depth of other encoding methods. All the representations in Table 1 uses a layer of single-qubit gates in the first step to prepare the superposition of pixel positions, which adds unit depth to the circuit. Following that, controlled gates are applied consecutively in time which encode the pixel values. Thus, the time depth of an encoding circuit mainly depends on the number of controlled gates used. From Table 1, it is clear that MCQI uses maximum number of gates (quadratic in pixel number), thus having maximum depth. The depth of NCQI and OCQR are comparable to each other, especially in the limit $n \gg 1$. As discussed in the last paragraph, HQDQR uses least number of elementary gates, so the time depth of HQDQR is minimum compared to the other three encoding methods.

### Image compression

The complexity of the encoding method, being linear in the number of pixels, becomes significantly high for high-resolution images. It is possible to drastically reduce the number of necessary gates for the encoding by using the minimization of logical functions. Here, the logical functions correspond to the bit values of pixel positions. This is a classical procedure, which has been previously discussed in^30,32,36^ for images encoded using only qubits or qutrits. For our proposed representation, however, both qubits and qutrits are used to encode the pixel positions. Hence, for the best-achieved compression, we must employ an algebra applicable on a combination of binary and ternary logical variables. In the following, we briefly explain the idea of image compression for a simple exemplary image.

**Figure 7:**
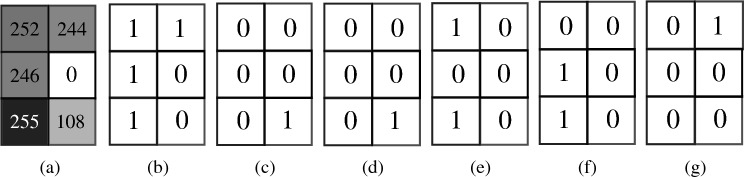
(**a**) A $2\times 3$ dimensional greyscale image with pixel values indicated on each pixel. (**b**, **c**) The six bitplanes of this image.

To start with, let us consider a $2\times 3$ greyscale image as shown in Fig. 7a. In Fig. 7b–g we show the 6 bitplanes corresponding to 6 qutrits in the first register, i.e. the $i^{\textrm{th}}$ bitplane shows the state of the $i^{\textrm{th}}$ qutrit for all the pixel positions. Let us denote the ternary states $\{0, 1, 2\}$ respectively by $\{x_{+}, x_{0}, x_{-}\}$, and the binary states $\{0, 1\}$ by $\{x, {\overline{x}}\}$. Now we consider Fig. 7b, in which all the pixels along the $1^{\textrm{st}}$ column has state $|1\rangle$ for the first qutrit. The pixel positions along this particular column are $\vert 00\rangle$, $\vert 10\rangle$ and $\vert 20\rangle$ respectively. It requires 3 generalized Toffoli gates to set the intensities of these three pixels. However, if we consider the sum *S* of the logical expressions of these pixel positions, and apply the ternary logic algebra^46^ as shown below,

15 $$\begin{aligned} S=x_{+}x + x_{0}x + x_{-}x =(x_{+}+x_{0}+x_{-})x =x, \end{aligned}$$

we see that instead of using three generalized Toffoli gates, we can encode these pixels by using a single controlled-*X* gate, where the control is only on the position qubit being in state $\vert 0\rangle$. Similarly, in the first row of Fig. 7b, the pixels positions are $\vert 00\rangle$ and $\vert 01\rangle$. We use the minimization of the logical expression as following

16 $$\begin{aligned} S^{\prime }=x_{+}x + x_{+}{\overline{x}}=x_{+}(x+{\overline{x}})=x_{+}, \end{aligned}$$

so that these two pixels can be encoded using one ternary controlled *X* gate, where the control is on the position qutrit being in state $\vert 0\rangle$. This idea of image compression for greyscale intensities is translated into RGB image by following the same procedure for each of the color channel intensities. The best achievable compression depends on both the image as well as the encoding method. For example, in^30^ the authors achieve $90.63\%$ compression, whereas in^32^ the compression obtained was $97.28\%$, and the compression obtained using qutrits in^36^ was $47.26\%$.

### Basic color image processing

In this section, we demonstrate some common RGB image operation using HQDQR encoding.

#### Channel swapping

The color channel swapping operation *CSO* is performed to swap the intensities of any two color channels. For our quantum image representation, it can be achieved by applying one of qutrit *X* gates $\{\sigma ^{x}_{01},\sigma ^{x}_{12},\sigma ^{x}_{02}\}$, whichever applies. For example, to swap the red and green channel, one needs to apply $\sigma ^{x}_{01}$ on the second register.

17 $$\begin{aligned}&CSO_{RG}|I\rangle = \frac{1}{\sqrt{2^{n}3^{m+1}}}\sum \limits _{Y=0}^{3^{m}}\sum \limits _{X=0}^{2^{n}} (|R_{XY}\rangle |1\rangle \nonumber \\& \qquad+ |G_{XY}\rangle |0\rangle + |B_{XY}\rangle |2\rangle ) \otimes |YX\rangle \end{aligned}$$

For swapping red and blue channel on the other hand, we have to apply $\sigma ^{x}_{02}$. The computational complexity of this operation is ${\mathscr {O}}(1)$.

**Figure 8:**
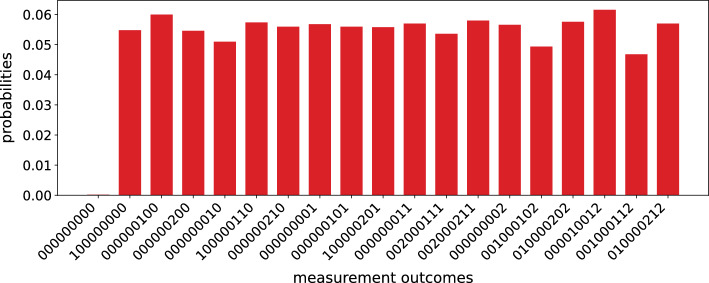
The probabilities of measurement outcomes of all the registers, when the circuit of Fig. 5 is simulated using Google Cirq qudit simulator. The data is collected for 5000 instances of measurements. The horizontal axis shows the basis states in the same order as in $\vert I\rangle$ in Fig. 4.

#### One channel operation

The one channel operation *OCO* performs a particular transformation to any one of the color channel intensities. For example, the red channel can be transformed by the following,

18 $$\begin{aligned}&OCO_{R}|I\rangle =\frac{1}{\sqrt{2^{n}3^{m+1}}}\sum \limits _{Y=0}^{3^{m}}\sum \limits _{X=0}^{2^{n}} (|R^{\prime }_{XY}\rangle |0\rangle + |G_{XY}\rangle |1\rangle \nonumber \\& \qquad+ |B_{XY}\rangle |2\rangle ) \otimes |YX\rangle , \end{aligned}$$

where $|R^{\prime }_{XY}\rangle =|R_{XY}^{\prime 5}..R_{XY}^{\prime 0}\rangle$ is the new intensity of the red channel. This is achieved by using a higher order hybrid Toffoli gate with $m+n+1$ control qudits and a maximum of six target qutrits.

## Experiment

To test our proposed image encoding in an ideal quantum system, we use Google Cirq’s quantum simulator, which provides an architecture of hybrid qudits and corresponding gates. The simulator is a classical simulator which mimics the behaviour of a quantum computer. We build the circuit of Fig. 5, and measure all the registers to retrieve the image state. The result is presented in Fig. 8a. The probability amplitudes of the resulting peaks fluctuate around the expected value $\big (\frac{1}{3\sqrt{2}}\big )^{2}\approx 0.0556$. This fluctuation may arise because of the random number generator used in the measurement constructor in Cirq. The state for which all the indiviadual qubits and qutrits are in state $\vert 0\rangle$ does not appear in $\vert I\rangle$, although it appears in the measurement outcome with negligible probability. We calculate the fidelity *F* of the quantum state vector generated from the simulated circuit with respect to $\vert I \rangle$ and get the value $F \approx 1$ in this case.

Next, we test the performance of the HQDQR image encoding circuit in presence of noise. We take into account two types of error that can occur in a quantum circuit. The first one is gate error which occurs due to the imperfect implementation of a gate. The other is idle error which occurs due to the decoherence and energy dissipation of a qudit because of its interaction with the environment. In our noise simulation, we model the gate error using depolarizing channel and the idle error using amplitude damping and phase damping channel.

*Depolarizing channel:* When a quantum state $\rho$ is subject to depolarizing channel, it evolves to a mixture of itself and other evolved states due to a number of different noise channels. For a qubit the most general effect of depolarizing channel can be expressed in the Kraus operator form as $\rho \rightarrow (1-p_{x}-p_{y}-p_{z})\rho + \frac{p_{x}}{3}\sigma _{x}\rho \sigma _{x}^{\dagger } + \frac{p_{y}}{3}\sigma _{y}\rho \sigma _{y}^{\dagger } + \frac{p_{z}}{3}\sigma _{z}\rho \sigma _{z}^{\dagger }$, where $p_{x}$, $p_{y}$ and $p_{z}$ are the strengths of bit-flip ($\sigma _{x}$), bit-phase flip ($\sigma _{y}$) and phase-flip ($\sigma _{z}$) channel respetively. We consider symmetric depolarizing channel for which $p_{x}=p_{y}=p_{z}$. For a qutrit depolarizing channel, the Kraus operators are Cartesian product of $\{\mathbb {I}_{3}, \sigma _{+1}^{x}, (\sigma _{+1}^{x})^{2}\}$ and $\{\mathbb {I}_{3}, z, z^{2}\}$ where

19 $$\begin{aligned} z = \begin{bmatrix} 1 &{} \quad 0 &{} \quad 0 \\ 0 &{} \quad e^{\frac{2\pi i}{3}} &{} \quad 0\\ 0 &{} \quad 0 &{} \quad e^{\frac{4\pi i}{3}} \end{bmatrix}. \end{aligned}$$

Thus the qutrit depolarizing channel is a combined effect of 8 different error channels. The two qutrit depolarizing channel Kraus operators can be obtained by taking the Cartesian product of the single-qutrit depolarizing channel Kraus operators. Similarly, the qubit-qutrit depolarizing channel is obtained from the Cartesian product of single-qubit and single-qutrit Kraus operators. More details on the qutrit depolarizing channel can be found in the paper by Gokhale et al.^44^.

*Amplitude damping channel:* The amplitude damping channel models the transition of a qudit from higher energy levels to lower energy levels. For a qubit, the only possible transition is $\vert 1\rangle \rightarrow \vert 0\rangle$ with decay probability $\lambda$. The Kraus operators for a qubit amplitude damping channel are

20 $$\begin{aligned} K_{0}= \begin{bmatrix} 1 &{} \quad 0 \\ 0 &{} \quad \sqrt{1-\lambda } \end{bmatrix}, \hspace{1em} K_{1} = \begin{bmatrix} 0 &{} \sqrt{\lambda } \\ 0 &{} 0 \end{bmatrix}. \end{aligned}$$

for a qutrit there is additional transition $\vert 2\rangle \rightarrow \vert 1\rangle$ with decay probability $\lambda _{21}$. We do not consider the transition $\vert 1\rangle \rightarrow \vert 0\rangle$ since it is comparatively suppressed is SC transmon qudits^47^, which is for example used in IBMQ processors. The Kraus operators of a qutrit amplitude damping channel are

21 $$\begin{aligned} K_{0}= \begin{bmatrix} 1 &{} \quad 0 &{} \quad 0\\ 0 &{} \quad \sqrt{1-\lambda _{10}} &{} \quad 0\\ 0 &{} \quad 0 &{} \quad \sqrt{1-\lambda _{21}} \end{bmatrix}, \hspace{0.5em} K_{1} = \begin{bmatrix} 0 &{} \quad \sqrt{\lambda _{10}} &{} \quad 0\\ 0 &{} \quad 0 &{} \quad 0\\ 0 &{} \quad 0 &{} \quad 0 \end{bmatrix}, \hspace{0.5em} K_{2}=\begin{bmatrix} 0 &{} \quad 0 &{} \quad 0\\ 0 &{} \quad 0 &{} \quad \sqrt{\lambda _{21}}\\ 0 &{} \quad 0 &{} \quad 0 \end{bmatrix}. \end{aligned}$$

*Phase damping channel:* Phase damping or dephasing is the main source of decoherence in a qudit. Due to phase damping the off-diagonal elements of the density matrix decays with time and eventually a pure state becomes a mixed state. The Kraus operators for a qubit Phase damping channel are

22 $$\begin{aligned} K_{0}= \begin{bmatrix} 1 &{} \quad 0\\ 0 &{} \quad \sqrt{1-\gamma } \end{bmatrix}, \hspace{0.5em} K_{1}= \begin{bmatrix} 0 &{} \quad 0\\ 0 &{} \sqrt{\gamma } \end{bmatrix}. \end{aligned}$$

The Kraus operators of a qutrit phase damping channel are the following.

23 $$\begin{aligned} K_{0}= \begin{bmatrix} 1 &{} \quad 0 &{} \quad 0\\ 0 &{} \quad \sqrt{1-\gamma } &{} \quad 0\\ 0 &{} \quad 0 &{} \quad \sqrt{1-\gamma } \end{bmatrix}, \hspace{0.5em} K_{1}= \begin{bmatrix} 0 &{} \quad 0 &{} \quad 0\\ 0 &{} \quad \sqrt{\gamma } &{} \quad 0\\ 0 &{} \quad 0 &{} \quad 0 \end{bmatrix}, \hspace{0.5em} K_{1}= \begin{bmatrix} 0 &{} \quad 0 &{} \quad 0\\ 0 &{} \quad 0 &{} \quad 0\\ 0 &{} \quad 0 &{} \quad \sqrt{\gamma } \end{bmatrix}. \end{aligned}$$

The dephasing rate $\gamma$ is different for a qubit and a qutrit.

The decay probabilities $\lambda _{ij}$s and the dephasing rate $\gamma$ depend on the gate time $\Delta t$ of single or two-qudit gates, the relaxaton time $T_{1}$ and pure dephasing time $T_{\phi }$ respectively according to the equations,

24 $$\begin{aligned}&\lambda _{ij} = 1 - e^{-\Delta t/ T_{1}^{ij}} \\ \nonumber&\gamma = 1 - e^{-\Delta t/ T_{\phi }}. \end{aligned}$$

Here $T_{1}^{ij}$ is the relaxation time between energy levels $\vert i\rangle$ and $\vert j\rangle$. In Table 2, we present the values of the noise parameters in the currently functional or experimental-level SC qudit quantum processors. In IBMQ qubit processors, both $T_{1}$ and total dephasing time $T_{2}$ varies in the wide range from several tens of $\mu \textrm{s}$ to 150 $\mu \textrm{s}$. We work with an intermediate value of $T_{1} = T_{2} \approx 100 \mu \textrm{s}$. Thus the pure dephasing time becomes $T_{\phi } = (\frac{1}{T_{2}} - \frac{1}{2T_{1}})^{-1} \approx 200 \mu \textrm{s}$. The single-qubit and two-qubit gate errors in IBMQ processors are of the order of $10^{-4}$ and $10^{-3}$ respectively. The $T_{1}$, $T_{2}$ and $\Delta t$ corresponding to qutrits are collected from the work by Blok et al.^47^ and the noise strength is calculated using those values. The gate errors corresponding to qutrits are collected from the paper by Morvan *et* al.^48^. Since we do not have the benchmarking data of a qubit-qutrit gate, we take the depolarizing error rate of these gates to be same as a two-qutrit gate.

**Table 2:** Different parameters of single-qudit and two-qudit gates in the current SC qudit quantum processors that we use for noise simulation.

Qudit	$T_{1}$ ($\mu$s)	$T_{2}$ ($\mu$s)	$T_{\phi }$ ($\mu$s)	Gate type	Gate time (ns)	$\lambda$	$\gamma$	Gate errors
Qubit	100	100	200	Single-qubit	100	0.001	0.0005	0.0001
				Two-qubit	300	0.003	0.0015	0.001
Qutrit	$T_{1}^{10}=$56, $T_{1}^{21}=$34.8	61.2	135	Single-qutrit	30	$\lambda _{10}=$ 0.0005, $\lambda _{21}=$ 0.0009	0.0002	0.001
				Two-qutrit	125	$\lambda _{10}=$ 0.002, $\lambda _{21}=$ 0.004	0.0009	0.1

Since we use only one qubit in the circuit of Fig. 5, the two-qubit gate errors are not put to use in our work. We take the gate error of a qubit-qutrit gate to be the same as the two-qutrit gate error.

Cirq provides in-built functions for applying the qubit noise channels, whereas the qubit-qutrit and qutrit noise channels are constructed by us in the Cirq framework using the Kraus operators. Before applying the error channels, we construct the circuit of Fig. 5 as a collection of Moments. In Cirq, a Moment is constituted of gates that act independently on different sets of qudits, thus they all act during the same abstract time slice. Each moment is then followed by application of gate errors corresponding to all the gates used in that moment, which is followed by application of idle errors on all the qudits. For more details on how the error channels are added to the circuit, please see the paper by Gokhale et al.^44^.

We consider the following two scenarios for our noise simulation.

1. All the noise parameters are chosen according to Table 2.
2. Anticipating the improvement of noise-robustness in near-future SC quantum processors, we reduce all the noise strengths by one order of magnitude. This means for qubits the single and two-qubit gate errors become respectively $10^{-5}$ and $10^{-4}$, $\lambda$ and $\gamma$ becomes respectively $10^{-4}$ and $10^{-5}$. Similar changes follow for the qutrit noise parameters.

**Figure 9:**
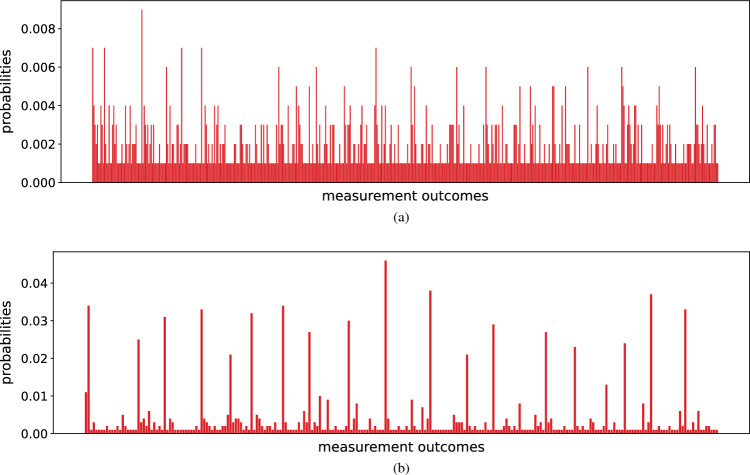
The probabilities of measurement outcome when the circuit of Fig. 5 is simulated in Cirq with added gate error and idle error. (**a**) The probabilities when the noise strength is chosen according to Table 2. (**b**) The probabilities when we lower all the noise strengths by one order of magnitude.

We present the results of the noise simulation for the above two scenarios in Fig. 9a and b respectively. From this, we see that the quantum image state preparation for the state-of-the-art SC qudit quantum circuit is highly inefficient. A large number of peaks emerge in the output. Some of the peaks are relatively higher than others, but not all of them correspond to the quantum image state $\vert I \rangle$. Looking at Table 2, the two-qutrit gate errors are highest which seems to contribute most to the noisy performance. The fidelity of the generated state is $F = 0.054$. However, when we consider the improved noise regime, in spite of appearence of additional peaks with small probabilities, the basis states corresponding to the quantum image state shows much higher probabilities. In this case, the image preparation remains plausible, and the fidelity is $F = 0.5377$.

It is crucial to check if the qubit-only image encoding methods perform better than HQDQR in the current SC qudit processors. For this, we compare HQDQR with OCQR. We choose OCQR over the other two encodings because MCQI has quadratic complexity, and NCQI requires 27 qubits to encode $$\vert I\rangle $$ which greatly slows down the noise simulation. If we choose noise parameters according to Table 2, the fidelity of OCQR is $$F_{OCQR} = 0.1865$$ which is higher than the corresponding HQDQR fidelity. Thus OCQR provides a better quantum image state preparation compared to HQDQR in current SC qudit processors. Again, the reason for this is the high two-qutrit gate errors. To verify this, we reduce two-qutrit gate errors to 0.01 keeping all other noise parameters unchanged. Now the fidelity for HQDQR becomes $$F =0.4793 $$ which is much better than OCQR.

Interestingly, single-qubit gate error of the order of $10^{-5}$, and $T_{1}$ and $T_{2}$ times of orders of milisecond have already been observed in experiments with SC qubits^49–51^. Also, the increasing interest in qutrit quantum computing implies that the improvement of qutrit quantum processors is imminent. Thus we can hope that the commercially available quantum processors in near future will achieve the improved noise regime we are considering here.

## Discussion

Quantum image processing is a promising venture towards achieving speed-up in image processing, which in turn is an indispensable task in a plethora of everyday applications. Standing in the era of NISQ devices, it is important to optimize the number of quantum units as well as the depth of a quantum circuit, in order to minimize the effect of noise in the output. In this work, we proposed Hybrid-qudit quantum representation (HQDQR) of RGB images, which uses only 7 qutrits to encode the information about color channels and their intensity. When compared with the existing encoding methods of RGB images with deterministic image retrieval, our representation uses least number of quantum units to encode the color information. Moreover, we considered both qubits and qutrits to encode the position information of the pixels, which can be an optimum choice while encoding a general rectangular image while keeping the number of redundant energy levels low. The complexity of HQDQR is polynomial in the number of pixels. We showed that HQDQR can be achieved by using much less number of elementary gates compared to the existing encoding methods, and it has the minimum circuit depth compared to them. The complexity can be further improved by using compression of logical expression corresponding to the pixel positions. Our noise simulation results show that in near-future SC quantum processors, HQDQR performs better than qubit-only image encoding methods. HQDQR naturally gives rise to a hybrid qubit-qutrit circuit. We demonstrate decomposition of higher order qubit-qutrit gates in terms of simpler single qudit and two-qudit gates in these systems. HQDQR can be used to check the performance of quantum convolutional neural networks for image classification. The current trend of research on higher dimensional quantum units and corresponding gates in these systems, indicates that quantum processing units including qudits will soon become available to users all over the world^38^, and thus our work will be a strong candidate for processing of RGB images.

## Data Availability

All data generated or analysed during this study are included in the manuscript.
